# Mental Health Impact of the COVID-19 Pandemic on Mexican Population: A Systematic Review

**DOI:** 10.3390/ijerph19116953

**Published:** 2022-06-06

**Authors:** Yazmín Hernández-Díaz, Alma Delia Genis-Mendoza, Miguel Ángel Ramos-Méndez, Isela Esther Juárez-Rojop, Carlos Alfonso Tovilla-Zárate, Thelma Beatriz González-Castro, María Lilia López-Narváez, Humberto Nicolini

**Affiliations:** 1 División Académica Multidisciplinaria de Jalpa de Méndez, Universidad Juárez Autónoma de Tabasco, Jalpa de Mendez 86205, Mexico; yazmin.hdez.diaz@gmail.com (Y.H.-D.); thelma.glez.castro@gmail.com (T.B.G.-C.); 2 Laboratorio de Genómica de Enfermedades Psiquiátricas y Neurodegenerativas, Instituto Nacional de Medicina Genómica, Mexico City 14610, Mexico; adgenis@inmegen.gob.mx; 3 División Académica de Ciencias de la Salud, Universidad Juárez Autónoma de Tabasco, Beautiful Villa 86100, Mexico; angel_mar@live.com.mx; 4 División Académica Multidisciplinaria de Comalcalco, Universidad Juárez Autónoma de Tabasco, Comalcalco 86040, Mexico; iselajuarezrojop@hotmail.com; 5 Hospital Chiapas “Dr. Jesús Gilberto Gómez Maza”, Tuxtla Gutierrez 29000, Mexico; dralilialonar@yahoo.com.mx

**Keywords:** depression, stress, anxiety, COVID-19, Mexico

## Abstract

The COVID-19 pandemic has had an impact on mental health in the general population, but no systematic synthesis of evidence of this effect has been undertaken for the Mexican population. Relevant studies were identified through the systematic search in five databases until December, 2021. The selection of studies and the evaluation of their methodological quality were performed in pairs. The Newcastle-Ottawa Scale (NOS) was used for study quality appraisal. The protocol of this systematic review was registered with PROSPERO (protocol ID: CRD42021278868). This review included 15 studies, which ranged from 252 to 9361 participants, with a total of 26,799 participants. The findings show that COVID-19 has an impact on the Mexican population’s mental health and is particularly associated with anxiety, depression, stress and distress. Females and younger age are risk factors for development mental health symptoms. Mitigating the negative effects of COVID-19 on mental health should be a public health priority in Mexico.

## 1. Introduction

Due to the current worldwide outbreak of corona virus disease 2019 (COVID-19), health systems are experiencing challenges in preventing infections, managing COVID-19 cases and ensuring effective strategies to protect the population [1]. Morbidity and mortality of COVID-19 around the world is higher in developing countries, including Mexico, than in developed countries [2]. Mexico has reported high death and case fatality rates due to COVID-19 since the first cases of SARS-CoV-2 virus infection reported at the end of February, 2020 [3]. 

At the onset of the pandemic, the Mexican government promoted preventive health behaviors including physical distancing, the use of hand sanitizer and staying at home. Additionally, some public health actions were enforced such as closing churches, schools, shopping centers and canceling mass events; however, these public health actions highly impacted the public economy [4].

The pandemic comes with a large number of potential stressors that might cause the mental health burden, such as mental disorders that need to be diligently addressed in the face of this crisis [5,6]. This context in adults usually has negative psychological effects and factors including boredom, fear of infection, frustration, lack of information and financial loss, and appears to increase the risk of mental health problems in the general population [7,8].

For example, Wang et al. [9] analyzed psychological responses during the initial stage of the pandemic in China. The authors found that 16.5% of the population reported depressive symptoms (moderate to severe), 28.8% reported anxiety symptoms (moderate to severe) and 53.8% of the population rated the psychological impact of the COVID-19 pandemic as severe.

The COVID-19 pandemic in Brazil is extremely severe: 70.3% of the adults have depressive symptoms and 67.2% have anxiety symptoms. Females and younger adults have a higher likelihood to present these symptoms [10]; meanwhile in France, being a woman can be considered a risk factor for avoidance mechanisms, intrusive thoughts, anxiety and stress [11].

The use of psychiatric diagnostic instruments in some countries and cultural settings may lead to either under- or over-enumerated mental disorders. It is important to identify differences in the prevalence of common mental disorders between regions or countries. To date, there is no systematic study about the mental health effects of the COVID-19 pandemic on the general population in Mexico. Therefore, it is necessary to address these points when designing mental health prevention/intervention programs. This systematic review will focus on articles on mental health and COVID-19 published after the outbreak of the COVID-19 pandemic in Mexico in terms of anxiety, depression, stress and distress. 

## 2. Materials and Methods

### 2.1. Design

Methods and results followed the recommendations of the Preferred Reporting Items for Systematic Reviews and Meta-Analyses (PRISMA) guidelines. The protocol of this systematic review was registered in the International Prospective Register of Systematic Reviews (PROSPERO) (protocol ID: CRD42021278868).

### 2.2. Search Strategy

Two authors (YHD and ADGM) independently identified records published up to 27 December 2021 through a literature search conducted in five databases: PubMed, EBSCO, Science Direct, PsychInfo and Scopus database. Published papers were searched using the following retrieval expressions: (COVID-19 OR SARS-CoV-2 OR severe acute respiratory syndrome coronavirus) AND (mental health OR depression OR anxiety OR stress) AND (general population OR general public OR public OR community) AND (Mexico).

### 2.3. Selection Criteria

We consider that papers included in the analysis should meet the following criteria: (1) assessed the mental health of the general population during the COVID-19 pandemic; (2) utilized standardized and validated scales for measurement; (3) age > 18 years; (4) English and Spanish written articles; (5) studies performed in the Mexican population. Studies were excluded if they: (1) focused on particular subgroups of the population (healthcare, children and adolescents, pregnant women and older adults); (2) did not include the Mexican population; (3) did not have full-text available. All possible papers went through a review process and duplicates were deleted. Then, abstracts and titles of the remaining papers were read for further assessment in order to determine if they were relevant for this study.

### 2.4. Data Extraction

One author (YHD) used a standardized form that the research team had developed to extract the details of the included studies. Data were extracted from each study, including (1) first author and year of publication, (2) the study design, (3) sample size, (4) sample characteristics, (5) assessment tools and (6) outcomes. A second author (ADGM) verified the extracted information and checked for accuracy and completeness. Differences were resolved through discussion.

### 2.5. Quality Appraisal

The Newcastle-Ottawa Scale (NOS) was used for study quality appraisal and adapted for cross-sectional studies, and was modified from the scale used in Epstein et al. [12]. The NOS scale was applied by two authors (YHD and ADGM), and any disagreements were resolved by the consultations with the third independent author (TBGC). The NOS scale appraises the methodological quality of three categories of studies: study selection, group comparability and exposure. A total of nine stars can be awarded following certain criteria, with a maximum of four stars for the selection category, a maximum of two stars for the comparability category and a maximum of three stars for the outcome category. Scores obtained in the NOS were then used to assign the study quality as good (>6 stars) or poor (<6 stars).

### 2.6. Data Synthesis

Data were analyzed according to the study outcomes and objectives. Descriptive (narrative) analyses of the included studies were conducted. As the studies had great heterogeneity in measuring and reporting mental health outcomes (anxiety, depression, stress and distress), conducting a meta-analysis was not possible. Therefore, findings were synthesized according to the reported outcomes.

## 3. Results

### 3.1. Search Results

**Figure 1:**
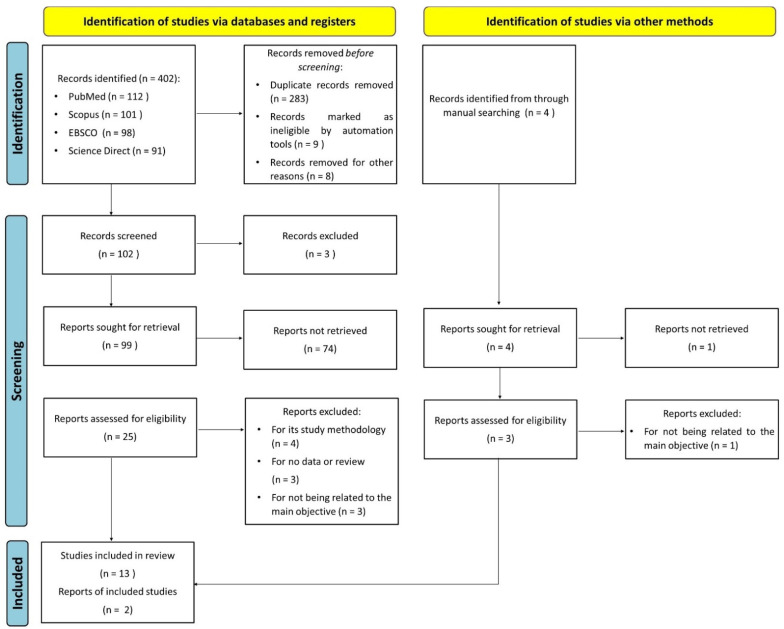
PRISMA flowchart of the inclusion process.

Figure 1 indicates the overall flow of the study selection, literature search and number of the included studies. In total, 406 publications were identified; we removed 283 duplicates. In total, 25 full-text articles were assessed for eligibility. There were 10 articles excluded due to their study methodology, not being related to the main objective, not having enough data or because they were reviews. After a meticulous review of each article, 15 studies met the inclusion criteria [13,14,15,16,17,18,19,20,21,22,23,24,25,26,27].

### 3.2. Methodological Characteristics of the Studies

Characteristics of the included studies are shown in Table 1. The sample size of the 15 studies included ranged from 252 to 9361 participants, with a total of 26,799 participants. All studies followed a cross-sectional study design. Data were collected online, inviting participants to fill in the questionnaires through social media platforms, emails and relevant groups or networks. To assess the effect of home confinement and social distancing resulting from the COVID-19 pandemic on the mental health of the general population, different scales and questionnaires were used, and these scales are summarized in Table 1. 

Generalized Anxiety Disorder-7 (GAD-7); State-Trait Anxiety Inventory (STAI); Patient Health Questionnaire (PHQ); Depression, Anxiety and Stress Scale (DASS-21); Beck Anxiety Inventory (BAI); and the Center for Epidemiologic Studies Depression Scale (CESD-R) were used for measuring anxiety or depression. Symptoms of stress were assessed using the Stress Coping Questionnaire (SCQ); COVID Stress Scale (CSS); the Perceived Stress Scale (PSS); the Fear of COVID-19 Scale (FCS); and the Questionnaire for the Detection of Risks to Mental Health COVID-19 (QDRMHC). Finally, the Questionnaire of Concerns and Behaviors related to COVID-19 (QCBC) and the Impact of Event Scale-Revised (IES-R) were used for assessing distress.

### 3.3. Quality Appraisal

Our quality assessment using the NOS scale for studies is presented in Table 2. The mean total score of NOS was 6.6. Therefore, all studies were identified as of good methodological quality due to the low risk of bias (scores > 6).

### 3.4. Symptoms of Anxiety and Associated Factors

Anxiety symptoms were analyzed in 11 out of the 15 studies [13,14,15,16,17,19,22,23,25,26,27], with a variation in the prevalence of anxiety symptoms from 11.9% to 93.7%. In Pérez-Cano et al. [13], 48.8% of individuals had anxiety from mild to very severe associated with the COVID-19 pandemic; moreover, 18.6% of these also experienced other symptoms such as stress from moderate to very severe. Of the total sample, 42% had anxiety as an emotional state and 26.8% showed high anxiety as a trait. 

Dosil-Santamaria et al. [23] indicated that people who have had the disease and those who have had someone close to them fall sick presented more anxiety than the rest. Additionally, only one study examined longitudinal differences in the severity of anxiety. Toledo-Fernández et al. indicated that anxiety symptoms did not change during the first months of the pandemic [14], and precautionary measures adopted to prevent the spread of COVID-19 could have had protective psychological effects for anxiety [16]. 

Finally, many factors were associated with higher levels of anxiety during the COVID-19 pandemic in Mexico, such as being female [13,16,19,22,23], younger age [16,22], history of direct or indirect contact with a COVID-19 confirmed case [16,23] and the presence of specific physical symptoms [16]. However, in some studies, no differences between genders or age concerning anxiety were identified [14,17,24,25,26,27].

### 3.5. Symptoms of Depression and Associated Factors

Symptoms of depression were analyzed in 9 out of the 11 studies [13,14,16,17,19,22,23,24,27]. The prevalence of depressive symptoms ranged from 5.2% to 86.6% in the Mexican population. The research by Terán-Pérez et al. [17] indicated that younger females reported a higher percentage of depression symptoms (26.4%) than older males (14.7%). Subjects who experienced depression also had moderate-to-very-severe anxiety or stress [13]. On the other hand, Cortés-Álvarez et al. [16] and Galindo-Vázquez et al. [19] found that divorced participants developed more depressive symptoms than single or married individuals.

Two studies examined longitudinal differences in the severity of depression. Loud et al. [24] indicated that depression was higher in July compared to March 2020; 19.9% of participants presented symptoms of depression in March, which increased to 27% in July. After adjusting for sociodemographic variables and smoking characteristics, all the participants were more likely to report symptoms of depression in July than at the beginning of the COVID-19 pandemic. However, Toledo-Fernández et al. [14] reported that depressive symptoms did not change during the first two months of the pandemic (5.22% and 6.26% for moderate to severe depression).

The main factors that contributed to having depression were being a female, divorced status, the presence of specific physical symptoms, a lack of confidence related to security of the test and a history of direct or indirect contact with a COVID-19 confirmed case [13,16,19,22,23]. However, precautionary measures taken by the general population to reduce the risk of contagion were associated with lower levels of depression [16,23]. 

### 3.6. Symptoms of Stress/Psychological Distress and Associated Factors

Of the total studies included, nine analyzed stress levels [13,16,18,20,23,24,25,26,27], and three evaluated psychological distress [14,16,21]. The prevalence of stress-related symptoms was >19%. Perez-Cano et al. [13] and González-Ramírez et al. [21] reported similar prevalence rates of stress (29.8%) and Post Traumatic Stress Disorder (PTSD; 27.7%), respectively. However, one study examined changes in perceived stress at two points during the pandemic (March and July 2020), reporting no statistically significant changes in the perceived stress. Additionally, it has been found that stress and PTSD vary according to sex, being higher in women [18,20,21]. 

Psychological distress was also assessed in three studies. One study reported a prevalence rate of symptoms of psychological distress at 50.3% [16], while Toledo-Fernández et al. [14] reported a prevalence of >20%; and finally, González-Ramírez [21] noted that the prevalence of psychological distress was 22% intrusive thoughts, 22.3% avoidance and 12.2% hyperarousal.

Some risk factors of stress symptoms also applied to symptoms of distress, including age (younger), sex (female), a perception of a high risk of contracting COVID-19, the number of people in the household, being in social isolation, changes in routine, engaging in less activity, loss of income and lower satisfaction of health information concerning COVID-19 [13,14,16,18,20,21].

## 4. Discussion

Our review explored the mental health status of the Mexican general population amid the COVID-19 pandemic. We performed a systematic search in five databases and included 15 studies for data extraction. The overall findings of this review suggest that a considerable proportion of the Mexican population has experienced mental health problems (anxiety, depression, stress and distress) during this COVID-19 outbreak.

Worldwide, several studies have been performed to explore the effects of the COVID-19 pandemic on the mental health of the general population. The COVID-19 pandemic has been associated with anxiety, distress, fear of contagion, insomnia and depression [28,29,30]. Prolonged social distancing protocols, economic stress and unemployment rates can originate a mental health crisis; moreover, social isolation has a detrimental effect on health, for both living alone and feelings of loneliness are risk factors for the increase in suicidal behavior [31]. 

The impact on mental health derived from the COVID-19 crisis may be severe in Mexico. Suicide and suicidal behaviors have continually increased in Mexico, and psychiatric conditions including anxiety and depression are associated with suicidal behavior [32,33]. An increase in suicide rates may become a significant public health issue in the country. Therefore, good mental health and prevention in the COVID-19 era are an important issue. More research studies are needed to understand how mental health consequences can be mitigated, hoping that the public health system efforts will reduce COVID-19-related suicides.

This review revealed the main factors that are associated with the development of anxiety, depression, stress and distress during the pandemic, with females and young adults being the most vulnerable. Similar findings have been observed in Turkey [34], Brazil [35], Peru [36] and China [37]. First, it has been suggested that women experience a high prevalence of stress and anxiety disorders which may contribute to the emergence or worsening of other symptoms such as eating disorders and PTSD [38,39]. Second, in terms of age, Mexican people under 40 years of age are in a more vulnerable position regarding their mental health status. For example, Ahmed et al. [40] revealed that young people (21–40 years) are in a more vulnerable position in terms of their mental health conditions and alcohol use. Glowacz et al. [41] indicted that those aged 18–30 years showed lower levels of occupational activity, less social contact and a poorer living environment, as well as higher levels of depression, anxiety and uncertainty than older adults. 

The current study provides coverage estimates for the prevalence data. Our systematic review included studies published since 2020, meaning that the ‘available data’ include prevalence studies published two years ago; therefore, changes in prevalence might occur. It is important to highlight that studies included in our systematic review presented several challenges. The search for the appropriate methodology due to the demographic, geographical and cultural differences of the various states of the country were important barriers to overcome while carrying out the studies. Moreover, the studies used scales that initially had been built for clinical use, but have proven their effectiveness for rapid screening and have been developed and validated for the Mexican population. For example, the PHQ scale has a sensibility and specificity of 88%, internal consistency (Cronbach a = 0.91) and test-retest reliability (interclass correlation, 0.83). It is important to highlight that these scales have the purpose of warning about the presence of symptoms; in no way can they be considered as a substitute for a clinical diagnosis.

Mental health in the post-COVID-19 era should not be ignored; this article lists the mental health problems more common in Mexico and identifies the main risk factors that are associated with anxiety, depression, stress and distress. This evidence should facilitate the development of programs based on the specific needs of the population to reduce the impact of the pandemic. That is to say, based on the known risk factors, it is possible to formulate evidence-based prevention and treatment strategies, so as to reduce the adverse psychological impact caused by the COVID-19 pandemic, for example, by (1) promoting mental health wellness and reducing distress; (2) establishing primary screening services for mental health; and (3) integrating basic mental health services into primary care for early detection.

This systematic review has a number of limitations. First, the comparability of studies and reliability of findings was further limited by the diversity of methods and tools used to assess depression, anxiety, stress and distress. Moreover, many studies failed to offer detailed information of the selected individuals or valid data on important factors. Therefore, we were not able to conduct a meta-analysis as there were many confounding factors that could affect the results, which need to be analyzed further. Second, participants in the included studies were recruited using an online questionnaire survey which is likely to have a sampling error. The data collected online may have created a selection bias and a lack of validity in the absence of face-to-face interviews; therefore, the findings are not generalizable to the whole population. Third, the studies only reflected the psychological state of the population at the time of the survey, and no long-term follow-up was conducted. Fourth, we excluded a number of studies from our analyses, due to a lack of and/or unclear information. Finally, we included only studies published in English and Spanish.

## 5. Conclusions

The findings from our systematic review suggest that a considerable proportion of the Mexican population has experienced mental health problems (anxiety, depression, stress and distress) during this COVID-19 outbreak. Thus, it is essential to develop psychological interventions that can improve the mental health of vulnerable groups and examine the long-term impact of the COVID-19 pandemic in the Mexican population.

## Figures and Tables

**Table 1:** Summary of included articles.

First Author and Publication Year	Sample Size (n)	Sample Characteristics	Survey Method	Assessment Tool	Emotions/Mental Health	Main Outcomes
Galindo-Vázquez, O. 2020 [19]	1508	Mean age: 34.4; Men: 385	Online questionnaire	- PHQ-9 - GAD-7	- Anxiety: 20.8% - Depression: 27.5%	Identified risk factors for depression and anxiety: female gender, single status, comorbidities and a history of mental health care.
Zamarripa, J. 2020 [18]	1173	Mean age: 25.9; Men: 522	Online questionnaire	- PSS	- Stress (M = 1.7; SD = 0.6)	The greater the number of weeks of social distancing, the higher the level of stress. Moreover, females have higher levels of stress than men.
Pérez-Cano, H. 2020 [13]	613	Mean age: 26.7; Men: 147	Online questionnaire	- DASS-21 - STAI	- Anxiety: 48.8% - Depression: 41.3% - Stress: 29.8%	Subjects with anxiety also had moderate to very severe depression or stress. Females have a higher proportion of anxiety, depression and stress than males.
Cortés-Álvarez, N.Y. 2020 [16]	1105	Age: >18; Men: 418	Online questionnaire	- IES-R - DASS-21	- Distress: 50.3% - Depression: 15.7% - Anxiety: 22.6% - Stress: 19.8%	Identified risk factors for distress, stress, depression and anxiety: female gender, divorced status, older age, lack of confidence related to security of the test and history of direct or indirect contact with a COVID-19 positive person. By contrast, precautionary measures were associated with lower levels of depression, distress, stress and anxiety.
González Ramírez, L.P. 2020 [21]	3932	Age: >18; Men: 1004	Online questionnaire	- IES-R	- PTSD: 27.7% - Distress: 22% (intrusive), 22.3% (avoidance) and 12.2% (hyperarousal)	Identified risk factors for distress: female gender, younger age, unemployed, single status, social isolation, change in routine, engaging in less activities and loss of income.
Gaeta, M.L. 2021 [15]	1290	Mean age: 24.2; Men: 384	Online self-report questionnaire	- SCQ	- Anxiety (M = 3.3; SD = 1.1)	Hope, tranquility, joy and gratitude, were positively related to self-regulated learning; although, disinterest and loneliness were negatively related.
Toledo-Fernández, A. 2021 [14]	670	Age: >18; Men: 280	Two-wave longitudinal online survey	- PHQ-9 - GAD-7 - QCBC	- Distress: 27.6% (waves 1) and 21.9% (waves 2) - Depression: 5.2% (waves 1) and 6.2% (waves 2) - Anxiety: 11.9% (waves 1) and 12.2% (waves 2)	Difference in distress levels were observed between the two waves. Moreover, a high-risk medical condition proved a considerable effect on distress.
Terán-Pérez, G. 2021 [17]	1230	Age: >18; Men: 366	Online questionnaire	- PHQ-9 - GAD-7 -	- Anxiety: 18.5% - Depression: 21.5%	No differences between genders and ages for anxiety and depression.
Medina-Fernández, I.A. 2021 [20]	956	Mean age: 21; Men: 213	Online questionnaire	- FCS - CSS	- Stress (M = 98.2; SD = 25.4)	Relationship was found of the age variable with fear, danger of contamination with the traumatic stress and fear with stress regarding COVID-19.
Morales-Chainé, S. 2021 [25]	9361	Mean age: 33; Men: 2668	Online questionnaire	- QDRMHC	- Anxiety (M = 40.1; SD = 31.8) - Stress (M = 38.5; SD = 25.6)	Avoidance, anger, sadness and withdrawal were associated with anxiety and acute stress.
Rodríguez-Hernández, C. 2021 [27]	1667	Mean age: 33.7; Men: 300	Online questionnaire	- DASS-21	- Anxiety (M = 7.1) - Stress (M = 9.7) - Depression (M = 9.7)	The symptom’s intensity was lower than expected, probably due to the high levels of resilience.
Loud, E.E. 2021 [24]	2753	Age: >18 Men: 1431	Online questionnaire	- PHQ-2	- Depression: 19.9% (waves 1) and 27% (waves 2) - Stress: M = 5.2; SD = 1.8 (waves 1) and M = 5.3; SD = 1.8 (waves 2)	Depression was higher in July compared to March, 2020; but the perceived stress did not change.
Ramírez-Dolores, C. 2022 [26]	316	Age: >18; Men: 144	Online questionnaire	- PSS-14 - BAI	- Anxiety (M = 14.3; SD = 11.5) - Stress (M = 26.2; SD = 7.9)	Influence of the built environment and climate on the levels of anxiety and stress were explored.
Dominguez-Rodriguez, A. 2022 [22]	5224	Age: >18; Men: 654	Online questionnaire	- GAD-7 - CESD-R	- Anxiety: 93.7% - Depression: 86.6%	Identified risk factors for anxiety and depression: female gender, younger age, unemployed, medication and have attempted suicide.
Dosil-Santamaria, M. 2022 [23]	252	Mean age: 21.1; Men: 86	Online questionnaire	- DASS-21	- Anxiety (M = 5.6; SD = 4.8) - Stress (M = 8.3; SD = 5.2) - Depression (M = 7.3; SD = 5.8)	Females had higher scoring of anxiety, stress and depression than males. Those who suffered from COVID-19 and those who had someone close to them that fell sick presented more anxiety, stress and depression.

Post Traumatic Stress Disorder (PTSD), Patient Health Questionnaire (PHQ), Generalized Anxiety Disorder-7 (GAD-7), The Perceived Stress Scale (PSS), Depression, Anxiety and Stress Scale (DASS-21), State-Trait Anxiety Inventory (STAI), Impact of Event Scale-Revised (IES-R), Stress Coping Questionnaire (SCQ), Questionnaire of Concerns and Behaviors related to COVID-19 (QCBC), Fear of COVID-19 Scale (FCS), COVID Stress Scale (CSS), Questionnaire for the Detection of Risks to Mental Health COVID-19 (QDRMHC), Beck Anxiety Inventory (BAI), Center for Epidemiologic Studies Depression Scale (CESD-R).

**Table 2:** Quality assessment of the studies included according to the modified Newcastle-Ottawa Scale (NOS). A total of nine stars can be awarded following certain criteria, with a maximum of four stars for the selection category, a maximum of two stars for the comparability category and a maximum of three stars for the outcome category.

Author	NOS Category			Assessment
	Selection	Comparability	Outcome	
Galindo-Vázquez, O. 2020 [19]	★ ★	★ ★	★ ★	Good
Zamarripa, J. 2020 [18]	★ ★ ★	★ ★	★ ★	Good
Pérez-Cano, H. 2020 [13]	★ ★	★ ★	★ ★	Good
Cortés-Álvarez, N.Y. 2020 [16]	★ ★	★ ★	★ ★ ★	Good
González Ramírez, L.P. 2020 [21]	★ ★ ★ ★	★ ★	★ ★	Good
Gaeta, M.L. 2021 [15]	★ ★ ★	★ ★	★ ★	Good
Toledo-Fernández, A. 2021 [14]	★ ★	★ ★	★ ★	Good
Terán-Pérez, G. 2021 [17]	★ ★	★ ★	★ ★ ★	Good
Medina-Fernández, I.A. 2021 [20]	★ ★	★ ★	★ ★	Good
Morales-Chainé, S. 2021 [25]	★ ★	★ ★	★ ★	Good
Rodríguez-Hernández, C. 2021 [27]	★ ★ ★	★ ★	★ ★	Good
Loud, E.E. 2021 [24]	★ ★	★ ★	★ ★	Good
Ramírez-Dolores, C. 2022 [26]	★ ★ ★ ★	★ ★	★ ★	Good
Dominguez-Rodriguez, A. 2022 [22]	★ ★	★ ★	★ ★	Good
Dosil-Santamaria, M. 2022 [23]	★ ★ ★	★ ★	★ ★	Good

## Data Availability

Data are contained within the article.
